# A likely pathogenic variant putatively affecting splicing of *PIGA* identified in a multiple congenital anomalies hypotonia‐seizures syndrome 2 (MCAHS2) family pedigree via whole‐exome sequencing

**DOI:** 10.1002/mgg3.428

**Published:** 2018-07-04

**Authors:** Junli Yang, Qiong Wang, Qingcui Zhuo, Huiling Tian, Wen Li, Fang Luo, Jinghui Zhang, Dan Bi, Jing Peng, Dong Zhou, Huawei Xin

**Affiliations:** ^1^ Department of Pediatrics Qilu Hospital of Shandong University Jinan China; ^2^ Institute for Biology and Medicine Wuhan University of Science and Technology Wuhan China; ^3^ Children Rehabilitation Center of Linyi Women and Children's Hospital Linyi China; ^4^ MyGenostics Inc. Beijing China; ^5^ School of Pharmacy Linyi University Linyi China

**Keywords:** glycosylphosphatidylinositol, GPI, IGD, inherited GPI deficiency, MCAHS2, multiple congenital anomalies hypotonia‐seizures syndrome 2, phosphatidylinositol glycan anchor biosynthesis class A, *PIGA*, PIGA deficiency, splicing defect, WES, whole‐exome sequencing

## Abstract

**Background:**

Glycosylphosphatidylinositol (GPI) anchoring is a special type of protein posttranslational modification, by which proteins with diverse function are attached to cell membrane through a covalent linkage between the protein and the glycolipid. Phosphatidylinositol glycan anchor biosynthesis class A (PIGA) is a key enzyme in GPI anchor biosynthesis, somatic mutations or genetic variants of which have been associated with paroxysmal nocturnal hemoglobinuria (PNH), or PIGA deficiency, respectively. More than 10 *PIGA* pathogenic or likely pathogenic variants have been reported in a wide spectrum of clinical syndromes of PIGA deficiency, including multiple congenital anomalies hypotonia‐seizures syndrome 2 (MCAHS2).

**Methods:**

Whole‐exome sequencing (WES) was performed on two trios, that is., the proband's family and his affected maternal cousin's family, from a nonconsanguineous Chinese family pedigree with hypotonia‐encephalopathy‐seizures disease history and putative X‐linked recessive inheritance. Sanger sequencing for *PIGA* variant was performed on affected members as well as unaffected members in the family pedigree to verify its familial segregation.

**Results:**

A novel likely pathogenic variant in *PIGA* was identified through comparative WES analysis of the two affected families. The single‐nucleotide substitution (NC_000023.9:g.15343279T>C) is located in intron 3 of the *PIGA* gene and within the splice acceptor consensus sequence (NM_002641.3:c.849‐5A>G). Even though we have not performed RNA studies, in silico tools predict that this intronic variant may alter normal splicing, causing a four base pair insertion which creates a frameshift and a premature stop codon at position 297 (NP_002632.1:p.(Arg283Serfs*15)). Sanger sequencing analysis of the extended family members confirmed the presence of the variant and its X‐linked inheritance.

**Conclusion:**

WES data analysis along with familial segregation of a rare intronic variant are suggestive of a diagnosis of X‐liked PIGA deficiency with clinical features of MCAHS2.

## 1 INTRODUCTION

The causative variants for a wide range of clinical infantile hypotonia and seizures syndromes remain to be identified. With the development of next‐generation DNA sequencing technology more new causal genes or variants for this broad neurological disorders have been identified, including a class of variants in glycosylphosphatidylinositol (GPI) anchor biosynthesis genes (Kinoshita, 2014; Ng & Freeze, 2015).

More than 150 proteins (about 1‐2% of total proteins) with diverse functions in human are attached to the cell membrane via the glycolipid GPI, namely GPI anchor. In contrast to the majority of cell surface proteins which associate with cell membrane via stretches of transmembrane hydrophobic amino acids, soluble GPI‐anchored proteins (GPI‐APs) are attached to the plasma membrane by forming a covalent linkage between their carboxyl‐terminus and the glycan moiety of the GPI glycolipid. The GPI anchor can convey additional properties to the attached proteins which may regulate protein sorting, trafficking and dynamics (Kinoshita, Fujita, & Maeda, 2008; Paulick & Bertozzi, 2008; Zurzolo & Simons, 2016), and this highly dynamic mode of cell surface expression plays essential roles in numerous biological processes including neuronal and embryonic development (Matas‐Rico, van Veen, & Moolenaar, 2017; Ng et al., 2012).

GPI anchoring is an evolutionarily conserved protein posttranslational modification mechanism, and at least 30 genes are involved in the GPI anchor biosynthesis process, including a family of phosphatidylinositol glycan anchor biosynthesis class (*PIG*) and post‐GPI attachment to proteins (*PGAP*) genes. Defects of GPI anchoring are detrimental, and at least 18 genes have been reported to be affected in the inherited GPI deficiency (IGD) diseases, which are associated with various neuronal and developmental abnormalities (Pagnamenta et al., 2017).

*PIGA* is an X‐linked *PIG* family gene encoding the catalytic subunit of the GPI‐GlcNAc transferase (GPI‐GnT) complex for the synthesis of the first GPI precursor, that is, GlcNAc‐PI (N‐acetylglucosaminyl phosphatidylinositol) (MIM 311770) (Kinoshita, 2014). *PIGA* gene function is essential for normal development and its gene knockout leads to embryonic lethality in mouse models (Kawagoe et al., 1996; Nozaki et al., 1999). In human, somatic mutations as well as genetic variants of *PIGA* have been associated with multiple disease conditions. Somatic *PIGA* mutations cause paroxysmal nocturnal hemoglobinuria (PNH) (MIM 300818), an acquired complement‐mediated hemolytic disease resulted from loss of GPI‐anchored CD55 and CD59 on erythrocytes (Takeda et al., 1993). Inherited pathogenic variants cause PIGA deficiency with a wide spectrum of phenotypes and neurological abnormalities, including multiple congenital anomalies hypotonia‐seizures syndrome 2 (MCAHS2) (MIM 300868), West syndrome, X‐linked intellectual disability (XLID), Ferro‐Cerebro‐Cutaneous syndrome (FCC), Simpson‐Golabi‐Behmel syndrome type 2 (SGBS2) and early‐onset (infantile) epileptic encephalopathy (EOEE/EIEE), *etc* (Belet et al., 2014; van der Crabben et al., 2014; Fauth et al., 2016; Johnston et al., 2012; Joshi et al., 2016; Kato et al., 2014; Kim et al., 2016; Olson et al., 2017; Swoboda et al., 2014; Tarailo‐Graovac et al., 2015). The pathogenic variants in these disease conditions were believed to cause partial loss of function, and the protein may retain residual enzymatic activities.

In this paper, we reported a novel likely pathogenic variant in *PIGA* through genetic analysis of a nonconsanguineous Chinese family pedigree with remarkable hypotonia‐encephalopathy‐seizures disease history and putative X‐linked recessive inheritance.

## 2 MATERIALS AND METHODS

### 2.1 Ethical compliance

This study was approved by Research Ethics Committee of Qilu Hospital, Shandong University, and informed consent was obtained from responsible persons and parents on behalf of junior study participants (under the age of 18 years).

### 2.2 Laboratory studies

Peripheral blood sample collected from the affected patient for metabolic workup was performed at the Central Laboratory of Qilu Hospital. Magnetic resonance imaging (MRI) was performed at the Imaging Center of Qilu Hospital. Peripheral blood samples were collected from affected and unaffected family members. DNAs were extracted from standard procedures.

### 2.3 Whole‐exome sequencing (WES) and Sanger sequencing

WES was performed on affected males (i.e., IV:4, proband, and IV:2, proband's maternal cousin) and parental samples of proband (i.e., III:8 and 9) as well as parental samples of proband's maternal cousin (III:3 & 4). WES was performed in MyGenostics Inc. according to previous studies (Zhang et al., 2017). DNAs from extended family members were analyzed by Sanger sequencing to verify familial segregation of *PIGA* variant (NM_002641.3:c.849‐5A>G) among affected family members (IV: 2 and 4) and unaffected family (pedigree) members (II:2, 3, 6 and 8; III: 2, 3, 4, 6, 7, 8, and 9; IV: 1). The sequencing region spans intron3‐exon4 junction of *PIGA* (NM_002641.3) gene locus, which was amplified with primers introduced in the literature (Iida et al., 1994).

### 2.4 Comparative WES data analysis

The reference human genome GRCh37/hg19 was used in the analysis of WES data. Following WES sequencing, the identified SNP and INDEL variants were filtered to exclude known polymorphisms (variants listed in 1000 Genomes (2015 Aug) with a minor allele frequency (MAF) >0.05). Next, the variants in the two affected male individuals were filtered according to the recessive inheritance model, and those autosomal variants of homozygosity in the parents, and X‐linked variants of homozygosity in the mothers or hemizygosity in the fathers were excluded. The remaining variants of the two affected male individuals were compared for variants in common. The common variants of homozygosity or hemizygosity in both affected male individuals, as well as those of compound heterozygosity after being filtered by SIFT, Polyphen‐2, and GeneTalk, were searched in the public variant databases for further pathogenicity predication and analysis (Kamphans et al., 2013).

### 2.5 In silico analysis of *PIGA* variant

In silico analysis of the intronic variant of *PIGA* gene (NM_002641.3:c.849‐5A>G) was performed using the following splice site prediction programs: HSF, Human Splicing Finder based on position weight matrices algorithm, MaxEntScan based on maximum entropy algorithm (http://www.umd.be/HSF3/index.html) and SplicePort (http://spliceport.cbcb.umd.edu) based on feature generation algorithm. The wild‐type and variant‐containing genomic sequences spanning exon3‐intron3‐exon4 region of *PIGA* gene were introduced to the programs to predict and compare the consensus values (CV scores) for the native and possible new splice acceptor site(s).

## 3 RESULTS

### 3.1 Clinical phenotypes

**Figure 1:**
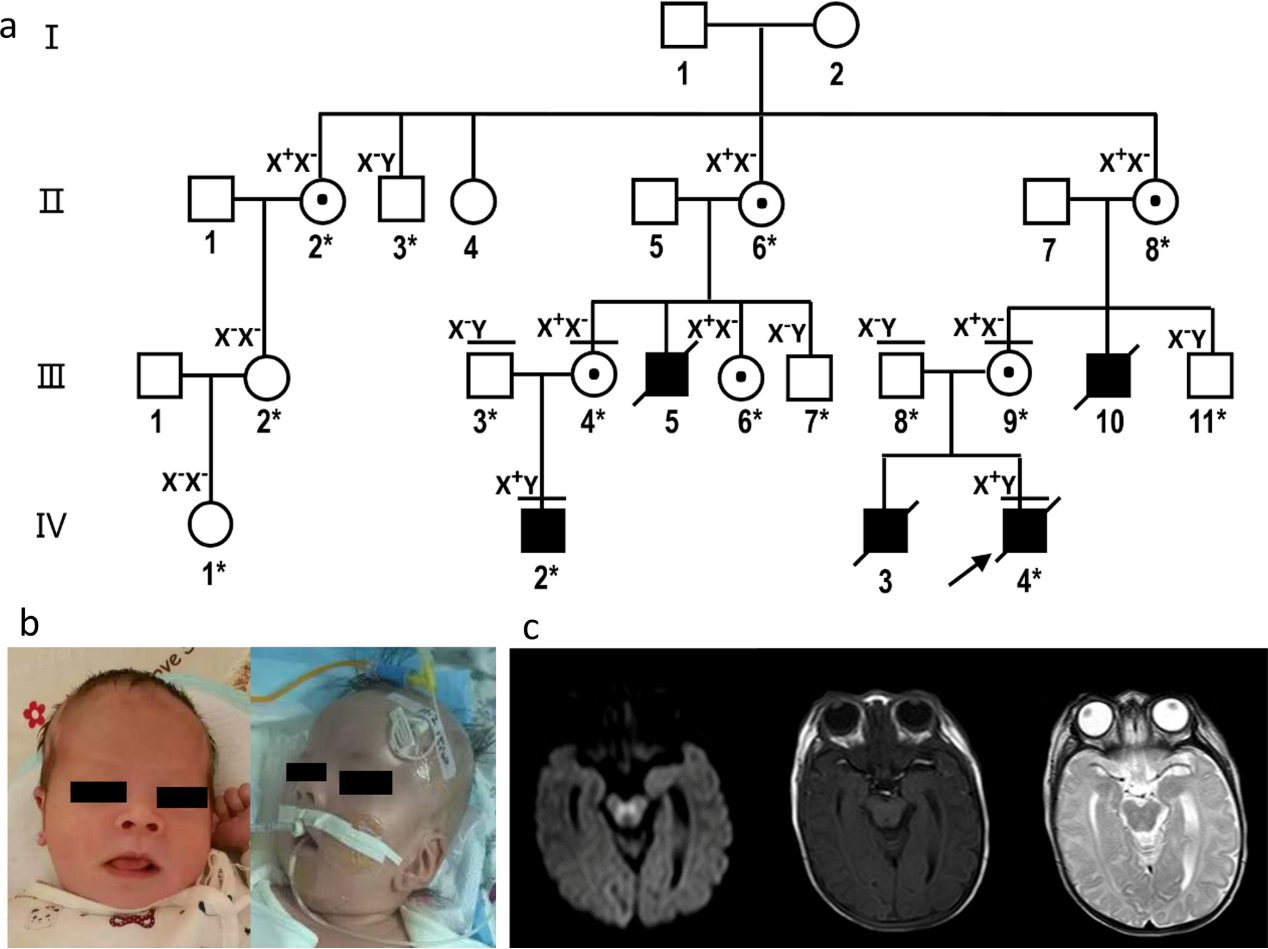
Family pedigree and patient images. (a) Schematic nonconsanguineous Chinese family pedigree with variant genotypes of *PIGA* (NM_002641.3). The family members for whom WES was performed are noted with a horizontal bar above their respective symbols, and the family members whose variant statuses were examined by Sanger sequencing are noted with an asterisk on the numerals. The variant statuses are represented as X+ for variant allele (NM_002641.3: c.849‐5A>G), and X‐ for wild‐type allele. The proband is shown with an arrow. (b) Facial images of the proband at the age of 15 days (left) and 30 days (right) after birth. Minor dysmorphism was observed showing a concave nasal bridge and low‐set ears. (c) Brain MRI image of the proband at the age of 30 days. Left, DWI, diffusion‐weighted image; middle, T1‐weighted image; right, T2‐weighted image. Brain abnormality was observed in DWI showing high symmetry flake signal in bilateral pontine tegmental area and testibrachium

A nonconsanguineous Chinese family pedigree was identified with remarkable hypotonia‐encephalopathy‐seizures disease history and putative X‐linked recessive inheritance (Figure 1a). The proband male patient (IV:4) was the second son of healthy parents (III: 8 and 9). The boy was born to the then 25‐year‐old mother (and 26‐year‐old father) with apparently normal measurements and physiological condition. His birth weight was 3400 g, length was 50.5 cm, and head circumference was 34 cm. At age of 15 days the boy showed basically normal facial expression (Figure 1b), as described. At age of 29 days, the boy was admitted to hospital (Qilu hospital, Jinan, Shandong Province, China) with psychomotor development delay, hypotonia, and encephalopathy features, including loss of facial expression and eye pursuit (Figure 1b). Blood tests showed mostly normal metabolism ranges, and largely uninformative. In accordance with the symptom of hypotonia and encephalopathy magnetic resonance imaging (MRI) showed brain abnormalities, including high symmetry flake signal in bilateral pontine tegmental area and testibrachium in diffusion‐weighted image (DWI) (Figure 1c). Dynamic electroencephalogram monitoring showed normal background wave, and no epilepsy wave was seen. Minor dysmorphism was observed. The nasal bridge was concave, and ears were low‐set. The patient died from respiratory complications at age of 2 months.

The proband had a brother (IV:3, first son of III:8 & 9) who was born 4 years earlier, and, as described, was apparently normal at birth. The brother had hypotonia at 3 months, and started repeated seizures (epilepsy) at 8 months with intellectual disability. The brother died at 1 year and 5 months old. The proband's mother (III:9), who was normal and healthy, had two siblings (brothers) (III:10 and 11). One brother (III:10), who was born 23 years ago, as described, had similar hypotonia, seizures and intellectual disability, and died at 1 year and 6 months old. The other brother (III:11) appeared normal and healthy, who was 20 years old at the time. The proband's grandparent of maternal side (II:7 and 8) were apparently normal, as described. The proband's maternal male cousin (IV:2) was 1 year and 5 months old at the time when the proband was admitted to the hospital. The cousin showed hypotonia after birth, as described, and had repeated seizures (epilepsy) and intellectual disability at 10 months. The parents (III:3 and 4) were normal and healthy, who were 25 and 26 years old, respectively. The mother (III:4) had three siblings, that is, two brothers and one sister. One of the two brothers (III:5), who was born over 20 years ago, as described, had similar hypotonia, epilepsy, and intellectual disability, and died at around 1 year old. The other brother (III:6), who was about 20 years old, appeared normal and healthy. The sister (III:7), who was about 15 years old, appeared normal and healthy. The grandparent of maternal side (II:5 and 6) of the affected cousin were normal, as described. The other members of the family pedigree, including the great grandparent (I:1 and 2), and the other three siblings of the grandmothers of the two affected boys (II:2, 3 and 4), as well as their offsprings (III:2 and IV:1) were mostly normal without related disorders.

### 3.2 Identification of a novel *PIGA* variant by comparative WES

Due to the remarkable disease history in the proband's family pedigree, a thorough genetic analysis was ordered in purpose of identifying the causative variant. WES was performed on genomic DNAs from the two affected male individuals (IV:2 and 4) and their unaffected parents (III:3 and 4, 8 and 9, respectively) (Figure 1a).

Totally 7528 and 7736 SNP or INDEL candidate variants in 2613 and 2588 genes were identified from WES data of the two affected male individuals, that is, proband (IV:4) and his maternal cousin (IV:2), respectively (Supporting Information Table S1). After filtering of known polymorphisms and those variants present in either parent as homozygosity or hemizygosity, the remaining variants of the two affected males (1094 and 1202, respectively) were compared, and 420 (one X‐linked and 419 autosomal) variants in common were obtained (Supporting Information Table S2). Except for the only X‐linked variant in *PIGA* (NC_000023.9:g.15343279T>C; NM_002641.3:c.849‐5A>G), which is of hemizygosity in both affected male individuals, no autosomal variant was found of homozygosity in both patients among the 419 shared autosomal variants. Further analysis of the shared autosomal heterozygous variants using SIFT, Polyphen‐2, and GeneTalk, and search of public variant databases for possible pathogenic compound heterozygous variant revealed no positive results (Supporting Information Table S2). Therefore, through comparative WES analysis we found only one shared, hemizygous variant in *PIGA* gene as candidate causative agent.

The identified *PIGA* variant was not found in the public variant databases such as Exome Variant Server (NHLBI Exome sequencing project/ESP), 1000 Genomes, Exome Aggregation Consortium (ExAC), The Genome Aggregation Database (gnomAD), dbSNP138, etc (Supporting Information Tables S2 and S3).

### 3.3 Confirmation of the *PIGA* variant by Sanger sequencing and *in silico* analysis

**Figure 2:**
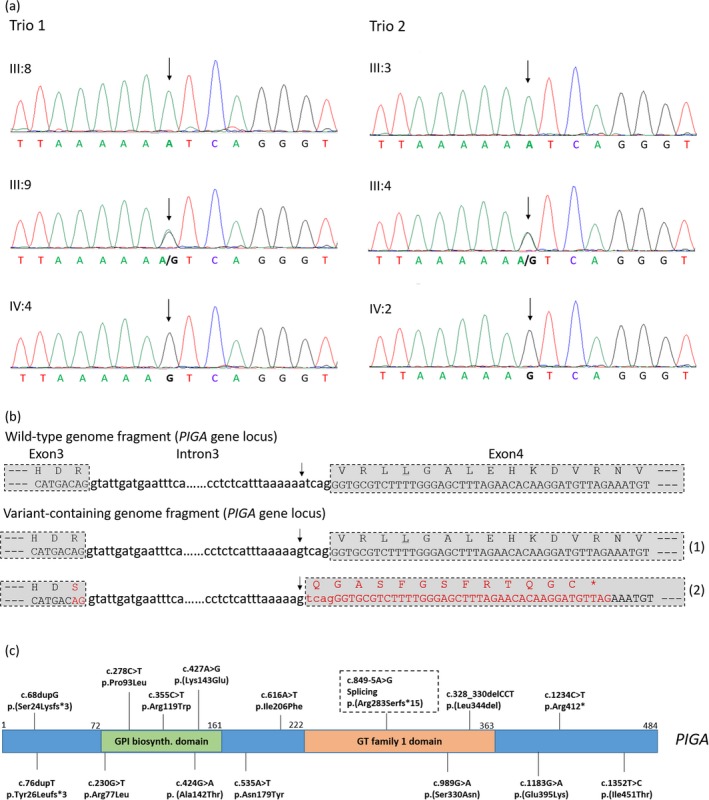
(a, b) Schematic illustration showing splicing alteration caused by the novel variant of *PIGA* (NM_002641.3:c.849‐5A>G). The arrow indicates the variant position. Two splicing modes are shown for the sequence containing the variant: (1) original (native) splice site; and (2) new (aberrant) splice site created by the variant, which led to frameshift and premature termination. (c) Schematic illustration of inherited *PIGA* variants including the one identified in this study. GPI biosynth. domain, GPI anchor biosynthesis domain; GT family 1 domain, glycosyltransferase family 1 domain. The PIGA protein reference sequence NP_002632.1 is used for variant nomenclature

The novel *PIGA* variant (NC_000023.9:g.15343279T>C; NM_002641.3:c.849‐5A>G) was confirmed by Sanger sequencing in the two affected families (Figure 2a). Both affected males (IV:2 & 4) carry the hemizygous variant. The two mothers (III:4 and 9) are heterozygous variant carriers, whereas the two fathers (III:3 and 8) are hemizygous wild‐type. Furthermore, the variant's X‐linked inheritance was also confirmed by Sanger sequencing of unaffected members of the family pedigree. No males tested (II:3, III:6 and 11) are hemizygous for this variant, and four females (II: 2, 6 and 8; III:7) were found heterozygous variant carriers (Figure 1a and Supporting Information Fig. S1).

The new *PIGA* variant (NM_002641.3:c.849‐5A>G) is located in intron 3, ‐5 bp to exon 4 splice acceptor site of the *PIGA* gene. As the blood samples were not available from the two affected male individuals for reanalyzing, no RT‐PCR was performed to examine the effect of the variant on the *PIGA* transcript. Instead we performed in silico analysis using the splice site prediction programs, that is, Human Splicing Finder (HSF), MaxEntScan and SplicePort. There is high probability that the variant may adversely affect the normal splicing, and, meanwhile, create a new splice acceptor site at ‐4 bp to the native site with higher consensus values (CV scores) (Supporting Information Table S4). The aberrant splicing may lead to a four base pair insertion, and cause frameshift at position 283 and a premature stop codon at position 297 (NP_002632.1:p.(Arg283Serfs*15)) (Figure 2b). The truncation disrupts the GT family 1 domain (glycosyltransferase family 1 domain) structure and deletion of C‐terminus of the protein, which putatively leads to loss of function (Figure 2c).

## 4 DISCUSSION

In this work we reported a clinical PIGA deficiency case of MCAHS2 phenotype in a nonconsanguineous Chinese family pedigree with hypotonia‐encephalopathy‐seizures disease history and putative X‐linked recessive inheritance. Through comparative WES analysis of two affected maternal cousin families we identified a single‐nucleotide intronic variant in the X‐linked *PIGA* gene (NC_000023.9:g.15343279T>C; NM_002641.3:c.849‐5A>G). The pathogenicity might arise from aberrant splicing of *PIGA* caused by the intronic variant, as indicated by *in silico* analysis, which may lead to frameshift and premature termination of protein translation (NP_002632.1:p.(Arg283Serfs*15)). Sanger sequencing of the patients’ families and extended members of the family pedigree confirmed the familial segregation of the X‐linked recessive *PIGA* variant. These genetic analysis results are suggestive of diagnosis of PIGA deficiency.

Next‐generation sequencing (NGS) has expedited the discovery of causative variants of IGDs with typical or atypical phenotypes. Up to now there have been at least 15 reported genetic variants of *PIGA* in clinical PIGA deficiency cases with most of which having been experimentally proven as pathogenic variants (Figure 2c). All these reported disease‐associated variants are located in the coding exons, including 10 missense variants at conserved positions of PIGA protein (NP_002632.1: p.Arg77Leu, p.Pro93Leu, p.Arg119Trp, p.(Ala142Thr), p.(Lys143Glu), p.Asn179Tyr, p.Ile206Phe, p.(Ser330Asn), p.(Glu395Lys), p.(Ile451Thr)), one in‐frame deletion (p.(Leu344del)), two duplications causing frame‐shifts (p.(Ser24Lysfs*3), p.Tyr26Leufs*3), and one non‐sense variant (p.Arg412*) (Figure 2b) (Fokstuen et al., 2016; Joshi et al., 2016; Kim et al., 2016; Olson et al., 2017; Soden et al., 2014; Zhu et al., 2015). In addition, there has also been one *de novo* missense variant (p.(Leu355Ser)) which has PIGA deficiency phenotype (Trump et al., 2016). In contrast, the novel variant we identified is not located in the coding exon as those previously reported, but in the intron region close to the splice acceptor site which may cause abnormal splicing. This is the first case of a probable splicing defective variant in the inherited PIGA deficiency cases.

Pathogenic variants affecting splicing are not rare in human diseases, and it has been estimated that about 15% or even more of genetic diseases caused by single‐nucleotide variants are due to splicing defects (Jian, Boerwinkle, & Liu, 2014). Somatic mutations in *PIGA* affecting splicing have been described in several PNH cases, in which the mutations reside mostly in the splice donor or receptor sites, and may severely affect or completely abolish native splicing, leading to exon skipping or evocation of cryptic splice sites (Maugard et al.,^1997^; Takeda et al., 1993). Genetic variants causing complete loss of function may not be retained in PIGA deficiency patients because only those which can retain partial protein production or function can survive and go through fetus development as inferred from gene knockout mouse models (Kawagoe et al., 1996; Nozaki et al., 1999). The variant identified in this study might not abolish the native splicing completely, and a certain low level of wild‐type mRNAs and proteins might be produced.

Splicing defective variants have been reported in several IGD cases other than PIGA deficiency. For example, at least three splicing defective variants of *PIGN* (MIM 606097), have been reported in patients of multiple congenital anomalies‐hypotonia‐seizures syndrome 1 (MCAHS1, MIM614080) and related syndromes (Fleming et al., 2016; McInerney‐Leo et al., 2016; Ohba et al., 2014). In particular, a lethal homozygous splicing defective variant in *PIGN* was identified in a fetus of syndromic congenital diaphragmatic hernia, which did not survive to birth mostly due to severe dysfunction of the protein (Brady et al., 2014). Also, at least three splicing defective variants of *PGAP1* (MIM 611655) have been reported in autosomal recessive mental retardation‐42 (MRT42, MIM 615802) and related syndromes with psychomotor retardation and brain atrophy as well as other features (Granzow et al., 2015; Kettwig et al., 2016; Novarino et al., 2014). More IGD cases with splicing defective variants include one variant of *PGAP3* (MIM 611801) in hyperphosphatasia with mental retardation syndrome 4 (HPMRS4, MIM 615716) (Knaus et al., 2016), two variants of *PIGL* (MIM 605947) in Chime syndrome (MIM 280000) and related neurodevelopmental disorder (Ng et al., 2012; Pagnamenta et al., 2017), one variant of *PIGO* (MIM 614730) in HPMRS2 (MIM 614749) (Krawitz et al., 2012), and one variant of *PIGG* (MIM 616918) in autosomal recessive mental retardation‐53 (MRT53, MIM 616917) (Makrythanasis et al., 2016), *etc*. Therefore, it is not quite surprising that a putative splicing defective variant was uncovered in *PIGA* gene under a disease condition.

The identification of the new causative variant of PIGA deficiency has attributed to a well‐preserved family pedigree which facilitates genetic analysis, that is, comparative WES and familial segregation analysis. As far as we know this is the first report of PIGA deficiency with familial inheritance in China. Recently there was a report of PIGA deficiency in a Chinese twin with a *de novo* variant in *PIGA* (Xie, Song, Li, & Jiang, 2018). Besides this there have been only a few sporadic IGD cases reported in China, including one MCAHS1 case caused by *PIGN* variants, and two Mabry syndrome (*i.e*., HPMRS1, MIM 239300 and HPMRS2) cases caused by *PIGV* (MIM 610274) and *PIGO* variants, respectively (Xu et al., 2017; Xue, Li, Zhang, & Yang, 2016). More *PIGA* or related IGD gene variants might be discovered in the future upon introduction of WES or NGS analysis of disease families in China that is enriched in big family pedigrees.

*PIGA* is the only X‐linked gene in the GPI biosynthesis pathway. All the currently described IGDs other than PIGA deficiency are mainly caused by autosomal homozygous or compound heterozygous variants in *PIG* or *PGAP* genes due to the recessive statuses of the variants (Kinoshita, 2014; Ng & Freeze, 2015). It is anticipated that, in contrast to these autosomal pathogenic variants, the pathogenic X‐linked *PIGA* variants might be more rarely retained in normal population. Indeed, a comparative search in the public variant databases of known pathogenic variants of *PIGA* and those of the other two MCAHS genes (*i.e*., *PIGN* and *PIGT* (MIM 610272) for MCAHS1 and MCAHS3 (MIM 615398), respectively), as an example, revealed that no pathogenic *PIGA* variants are found in the normal population, whereas most *PIGN* or *PIGT* pathogenic variants are found in normal population at low frequency (data not shown). That our variant of *PIGA* has not been found in normal population is suggestive of its pathogenicity.

PIGA deficiency has multiple clinical manifestations with developmental and neurological abnormalities. The patients in this study showed features mostly matched to those of MCAHS2 (van der Crabben et al., 2014; Johnston et al., 2012; Kato et al., 2014; Tarailo‐Graovac et al., 2015), and may be classified into a less severe type of PIGA deficiency, which did not show severe congenital anomalies (Tarailo‐Graovac et al., 2015). The phenotypes of our patients also showed certain similarity to MCAHS1 and MCAHS3 (Khayat et al., 2016; Nakagawa et al., 2016; Ohba et al., 2014). However, no pathogenic variants of *PIGN* or *PIGT* genes have been found in our patients.

The genetic analysis in this study is critical for disease management. PIGA deficiency patients may be responsive to ketogenic diet treatment, as a recent study showed (Joshi et al., 2016). As many genes are associated with epileptic encephalopathy and hypotonia besides *PIGA* and related GPI biosynthesis gene, and there have been a wide range of medicine in clinical use with many of which being directed to a particular genotype (e.g., quinidine is a specific medicine for *KCNT1* epileptic encephalopathy genotype) (Fukuoka et al., 2017), a solid genotype determination is needed to guide the treatment. Genetic analysis can also help guide the prenatal diagnosis, and help prevent the birth of PIGA deficient offspring in the future as an important precautious measure in family plan for the patient family and other members in the pedigree.

The major limitation of this work is that the effect of the novel variant on splicing of *PIGA* has not been experimentally tested. There was unavailability of patient sample for further mRNA/cDNA analysis. Instead, we performed a thorough *in silico* splice site prediction analysis for possible splicing alterations. Several *in silico* analysis tools were employed to predict the possible splicing defect based on different algorithms (*i.e*., Human Splicing Finder (HSF), MaxEntScan and SplicePort). All these *in silico* tools showed that the variant might create a new splice site with high probability, whereas the native splice site would be adversely affected. Especially the MaxEntScan program showed significant confidence on the prediction of the splicing alteration with a more than twofold increase in probability in the creation of the new splice site, and more than onefold decrease in probability in the retention of the native splice site, which strongly supported the prediction of the splicing defect caused by the variant.

In conclusion, we have identified a novel likely pathogenic variant in the *PIGA* gene in a clinical PIGA deficiency case of MCAHS2 phenotype. Although further work is still needed to confirm the splicing defect caused by the variant, genetic analysis as well as *in silico* analysis results supported the proposal of the novel variant in *PIGA* as the causative agent of the clinical case. A variant site‐specific gene‐edited cell or animal model would be helpful in the confirmation of the putative splicing defect, and is highly suggested for further study.
