# Supplementary figures and images for: A likely pathogenic variant putatively affecting splicing of PIGA identified in a multiple congenital anomalies hypotonia‐seizures syndrome 2 (MCAHS2) family pedigree via whole‐exome sequencing
Source: Mol Genet Genomic Med. 2018 Jul 4;6(5):739–48. doi: 10.1002/mgg3.428 (PMC6160699; doi:10.1002/mgg3.428)

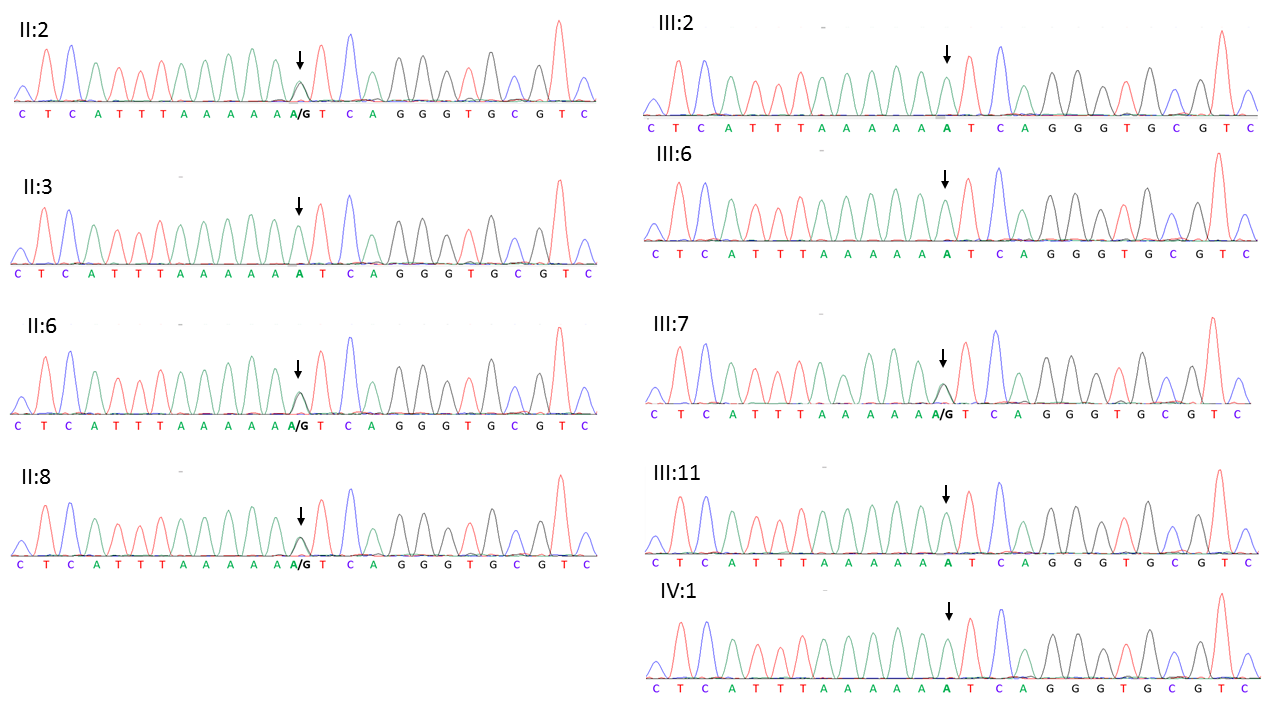

Supplement: Supplementary file 1 [file MGG3-6-739-s001.tif]
