# Supplementary material for: A likely pathogenic variant putatively affecting splicing of PIGA identified in a multiple congenital anomalies hypotonia‐seizures syndrome 2 (MCAHS2) family pedigree via whole‐exome sequencing
Source: Mol Genet Genomic Med. 2018 Jul 4;6(5):739–48. doi: 10.1002/mgg3.428 (PMC6160699; doi:10.1002/mgg3.428)
Supplement: Supplementary file 5 [file MGG3-6-739-s005.docx]

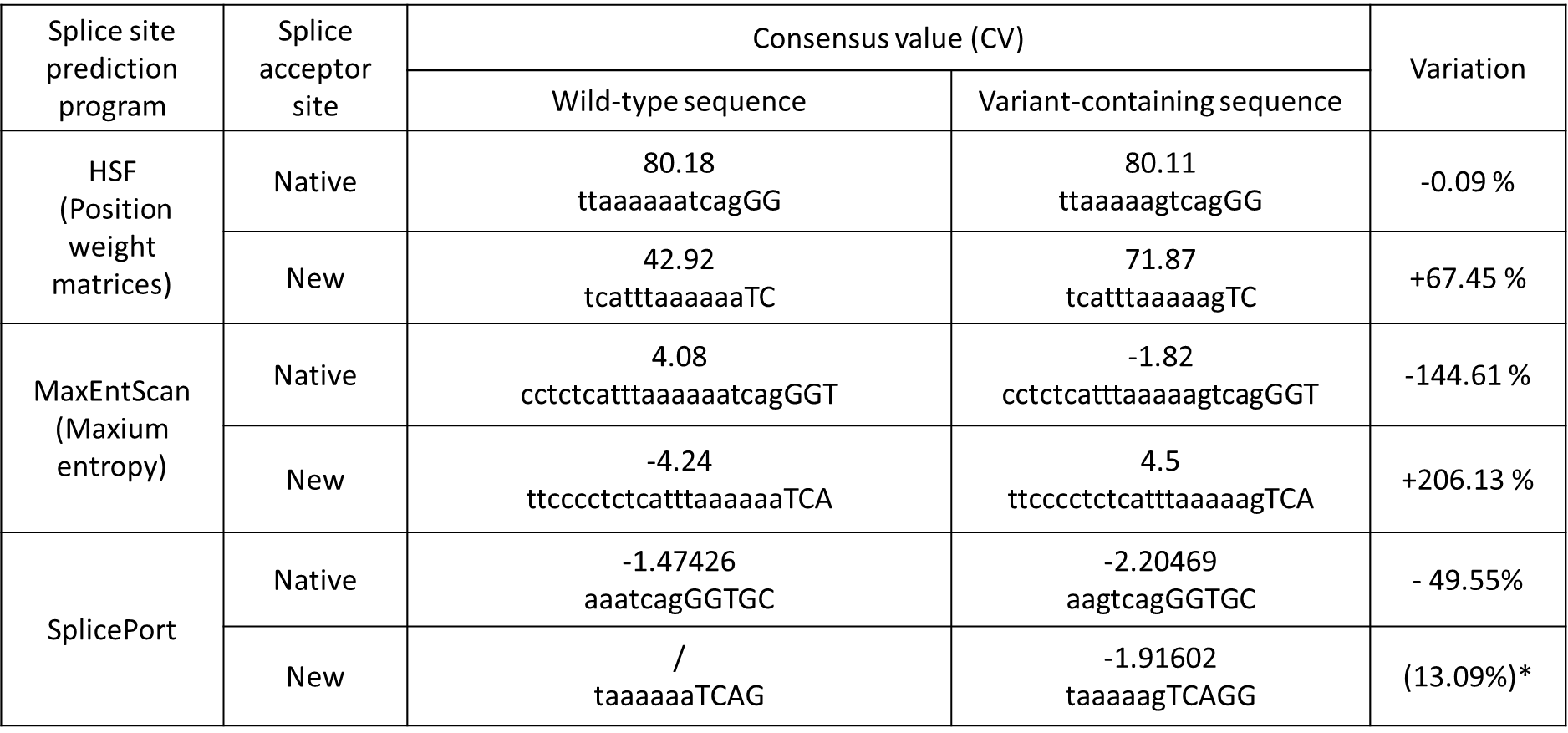


Supplementary Table 4. Summary of *in silico* splice site prediction analysis. Splice site prediction programs include HSF (Human splicing Finder), MaxEntScan, and SplicePort. The variations are shown as percentages. *This variation value is obtained by comparing the CV score of the new splice site to that of the native site with the variant sequence. The SplicePort program predicts no alternative splice site in the wild-type sequence.
